# Reducing tumor invasiveness by ramucirumab and TGF‐β receptor kinase inhibitor in a diffuse‐type gastric cancer patient‐derived cell model

**DOI:** 10.1002/cam4.4259

**Published:** 2021-09-20

**Authors:** Song‐Yi Lee, Seonggyu Byeon, Jihoon Ko, Sujin Hyung, In‐Kyoung Lee, Noo Li Jeon, Jung Yong Hong, Seung Tae Kim, Se Hoon Park, Jeeyun Lee

**Affiliations:** ^1^ Division of Hematology‐Oncology Department of Medicine Samsung Medical Center Sungkyunkwan University Seoul Korea; ^2^ Department of Internal Medicine Chungbuk National University Hospital Chungbuk National University College of Medicine Cheongju Korea; ^3^ Department of Mechanical Engineering Seoul National University Seoul Korea; ^4^ Department of Intelligent Precision Healthcare Convergence Sungkyunkwan University Suwon Korea

**Keywords:** angiogenesis, epithelial–mesenchymal transition (EMT), gastric cancer, ramucirumab

## Abstract

**Background:**

Diffuse‐type gastric cancer (GC) is known to be more aggressive and relatively resistant to conventional chemotherapy. Hence, more optimized treatment strategy is urgently needed in diffuse‐type GC.

**Methods:**

Using a panel of 10 GC cell lines and 3 GC patient‐derived cells (PDCs), we identified cell lines with high EMTness which is a distinct feature for diffuse‐type GC. We treated GC cells with high EMTness with ramucirumab alone, TGF‐β receptor kinase inhibitor (TEW‐7197) alone, or in combination to investigate the drug's effects on invasiveness, spheroid formation, EMT marker expression, and tumor‐induced angiogenesis using a spheroid‐on‐a‐chip model.

**Results:**

Both TEW‐7197 and ramucirumab treatments profoundly decreased invasiveness of EMT‐high cell lines and PDCs. With a 3D tumor spheroid‐on‐a‐chip, we identified versatile influence of co‐treatment on cancer cell‐induced blood vessel formation as well as on EMT progression in tumor spheroids. The 3D tumor spheroid‐on‐a‐chip demonstrated that TEW‐7197 + ramucirumab combination significantly decreased PDC‐induced vessel formation.

**Conclusions:**

In this study, we showed TEW‐7197 and ramucirumab considerably decreased invasiveness, thus EMTness in a panel of diffuse‐type GC cell lines including GC PDCs. Taken together, we confirmed that combination of TEW‐7197 and ramucirumab reduced tumor spheroid and GC PDC‐induced blood vessel formation concomitantly in the spheroid‐on‐a‐chip model.

## Graphical abstract

Diffuse‐type gastric cancer (GC) is known to be more aggressive and relatively resistant to conventional chemotherapy. In this study, we sought a drug candidate for this type of cancer treatment and confirmed that combination of TEW‐7197 and ramucirumab reduced tumor spheroid invasiveness and GC PDC‐induced blood vessel formation concomitantly in the spheroid‐on‐a‐chip model.

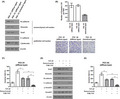

## 1 INTRODUCTION

Gastric cancer (GC) is the third leading cause of cancer‐related death in global health.^1^ Although declining *H*. *pylori* prevalence and active GC screening and surveillance using endoscopy have resulted in reduced GC incidence and mortality,^2^, ^3^, ^4^ many GC patients are initially diagnosed when they are in an advanced stage or incurable. A limited number of therapeutic options, the resistance of chemotherapy, and cancer heterogeneity are a significant hurdle for GC treatment.

Diffuse‐type GC by Lauren classification, is often originated from signet ring cell carcinoma. Compared to intestinal type, it has distinct clinicopathologic features; those are young age at diagnosis, no pre‐malignant lesions, loss of E‐cadherin, presence of peritoneal seedings, and worse prognosis.^5^ In The Cancer Genome Atlas (TCGA) project and Asian Cancer Research Group (ACRG) data, the distinct cluster of diffuse‐type GC was confirmed by molecular subtypes as genomic stable (GS) and microsatellite stable with epithelial to mesenchymal transition features (MSS/EMT), respectively.^6^, ^7^ In GS subtype, mutations in *CDH1* (which encodes E‐cadherin) and *RHOA* (RhoA) were detected relatively often (37% and 15%, respectively), which possibily caused invasive phenotypes of diffuse‐type GC.^6^

The EMT is a process by which epithelial cells escape from their normal stationary status and acquire cell plasticity that is typically characteristic of mesenchymal cells.^8^ Emerging evidences have suggested that EMT contributes to chemoresistance that results from multiple drug resistance (MDR) induced by the overexpression of drug resistance‐related genes.^9^, ^10^, ^11^ Because cytotoxic chemotherapy is the first‐line systemic treatment of advanced GC,^12^ this could explain the aforementioned poor consequence of diffuse‐type GC patients. While the importance of targeting the EMT in cancer treatment has been well recognized, but few strategies to overcome EMT‐related chemoresistance have been developed. As a result, there are strong unmet needs for therapeutic interventions targeting the EMT to improve outcomes in patients with diffuse‐type cancers.

Ramucirumab is a monoclonal antibody targeting vascular endothelial growth factor (VEGF) receptor 2. Widely used as a single agent or combined treatment for advanced GC, it has been proven to show improved outcomes when applied with chemotherapy like paclitaxel by relieving chemotherapy‐induced resistance.^13^, ^14^

In this study, we attempted to demonstrate the potential effect of ramucirumab and TGF‐β receptor inhibitor on tumor invasiveness and PDC‐induced angiogenesis in diffuse‐type GC cell line and PCD models. In order to visualize the tumor‐induced angiogenesis, we established a 3D tumor spheroid‐on‐a‐chip using PDC and observed variations in tumor‐induced angiogenesis on a chip upon different drug treatments.

## 2 MATERIALS AND METHODS

### 2.1 Cell culture

Human GC cell lines were purchased from the Korea Cell Line Bank (Hs‐746T, MKN1, MKN74, N87, SNU216, SNU484, and SNU668 cell lines) or the American Type Culture Collection (AGS, MKN28, and MKN45 cell lines) in 2010. All cell lines were cultured in RPMI 1640 medium supplemented with 10% FBS, an antibiotic, and an antimycotic (Invitrogen Corporation) at 37℃ in a humidified 5% CO_2_ atmosphere. Human umbilical vein endothelial cell (HUVEC; Lonza) was cultured in endothelial growth medium (EGM‐2; Lonza) and passages 4–5 were used for experiments. Lung fibroblast (LF; Lonza) was cultured in fibroblast growth medium (FGM‐2; Lonza) and passages 5–6 were used. HUVEC and LF were incubated at 37℃ in 5% CO_2_ for 2–3 days prior to chip loading.

All cell lines were tested for mycoplasma contamination and authenticated by STR DNA profiling (Samsung Medical Center Basic Research Support Center) for every 6 months, and all cells were cultured according to the manufacturer's instructions and had been passaged for fewer than 2 months after thawing.

### 2.2 Patient‐derived cell line culture

The GC patients were enrolled as part of the SMC Oncology Biomarker study (NCT#01831609, clinicaltrials.gov), which is reported elsewhere. Effusions or tumor tissues were collected for therapeutic purposes after obtaining written informed consent, and all procedures were performed according to the Declaration of Helsinki guidelines. The Institutional Review Board at Samsung Medical Center approved the protocol.

Primary biopsies or ascites fluid from patients with advanced GC were cultured as previously reported.^15^, ^16^ Cells were cultured in RPMI 1640 medium supplemented with 10% fetal bovine serum, 1% antibiotic‐antimycotic solution (Gibco BRL), 0.5 μg/ml of hydrocortisone (Sigma Aldrich), 5 μg/ml of insulin (PeproTech), and 5 ng/ml of epidermal growth factor (PeproTech). The culture medium was changed every 3 days, and the cells were maintained at 37℃ in a humidified 5% CO_2_ incubator. The PDCs were passaged using TrypLE Express (Gibco BRL) to detach the cells when the cultures reached 80%–90% confluence.

### 2.3 Western blot analyses

Total cell extracts were obtained using lysis buffer (20 mM HEPES [pH 7.4], 1% Triton X‐100, 1 mM EDTA, 1 mM MgCl_2_, 150 mM NaCl, 10% glycerol, protease inhibitor, and phosphatase inhibitor cocktail [Invitrogen]) and the protein concentration was determined using the micro‐BCA protein reagent (Pierce Biotechnology). Equal amounts of protein (30 μg) from the clarified lysates were separated by SDS‐PAGE and transferred onto nitrocellulose membranes having a 0.2 μm pore size (Whatman). The membranes were incubated in antibodies against N‐cadherin (#13116, 1:1000; Cell Signaling Technology [CST]), E‐cadherin (#14472, 1:1000; CST), vimentin (#5741, 1:1000; CST), SNAIL (#3879, 1:1000; CST), VEGFR2 (#9698, 1:1000; CST), Smad2/3 (#3102, 1:1000; CST), phosphor‐Smad2 (Ser 465/467)/Smad3 (Ser 423/425) (#8828, 1:1000; CST), β‐catenin (#8480, 1:1000; CST), cytokeratin (ab9023, 1:2000; Abcam), and β‐actin (AC‐15, 1:5000; Sigma) An ECL system was used for protein detection (Invitrogen).

### 2.4 Invasion assay

Cell invasiveness was measured using Matrigel‐precoated Transwell plates (Corning Costar). Cells were incubated with a stem cell signaling pathway inhibitor for 48 h, trypsinized, counted, and added to the upper chambers of the Transwell plates. Medium containing 10% FBS was added to the lower chambers and acted as the chemoattractant. After incubation for 24 h, cells in the lower chamber were fixed in 100% methanol and stained with 0.1% crystal violet. Cells were counted in 10 randomly selected areas of each well using microscopy. Data were expressed as the mean ± SD from three independent experiments. Unless data were shown as the number of cells, the percentile values were calculated by dividing the number of drug‐treated cells of each group with the number of non‐treated control cells.

### 2.5 Spheroid formation assay

When the cultures reached 80% confluence, the cells were detached with trypsin–EDTA and re‐plated at a density of 5 × 10^3^ cells/well in ultra‐low attachment 6‐well plates (Corning) containing serum‐free DMEM/F12 medium supplemented with 20 ng/ml of epidermal growth factor (PeproTech), 20 ng/ml of basic fibroblast growth factor (PeproTech), and 2% of B27 supplement (Invitrogen).

After 2 weeks of incubation, spheroids greater than 50 μm in diameter were counted under a phase‐contrast microscope at 10× magnification. The average number of spheroids per 5000 seeded cells was calculated from three independent experiments. Unless data were shown as the number of cells, the percentile values were calculated by dividing the number of drug‐treated cells of each group with the number of non‐treated control cells.

### 2.6 Spheroid formation for 3D angiogenesis assay

Similarly, as in the previous step, PDCs and lung fibroblast were cultured until confluent and then re‐suspended at 8000/ml and 2000/ml, respectively. The suspension was mixed with a 1% volume ratio of Matrigel and dispensed 10,000 cells per well. The PDC suspension was incubated in the U‐shaped 96‐well plate (Sumitomo Bakelite) for 2 days for spheroid formation.

### 2.7 PDC spheroid‐induced 3D angiogenesis assay

The loading procedure of tumor spheroid‐on‐a‐chip follows a previously published article.^17^ The fibrin hydrogel containing PDC spheroid and 1 mi/ml LF was loaded onto the chip. After the hydrogel cross‐linked, 30 μl of the cell suspension (10^6^/ml of HUVEC) was exposed adjacent to the primed hydrogel interface. After then, the media reservoir was filled with 200 μl of the medium. The samples were incubated for 4 days and the culture medium was replaced every 2 days.

### 2.8 Drug treatment for 3D angiogenesis model

For the monoclonal antibody, ramucirumab was diluted at 2 μM with EGM‐2. TEW‐7197 was dissolved in DMSO at 1 μM according to the manufacturers’ instructions. The drugs were introduced into the sample at the target concentration along with the culture medium on Day 2.

### 2.9 Immunocytochemistry and image acquisition

The samples were fixed with 4% (w/v) paraformaldehyde (Biosesang) in DPBS (WELGENE) for 15 min, followed by permeabilization with a 20 min immersion in 0.20% Triton X‐100 (Sigma). The samples were then treated with 3% BSA (Sigma) for 24 h. Endothelial cell (EC)‐specific staining was performed using 488 fluorescein‐labeled Ulex Europaeus Agglutinin I (Lectin; Vector), which was prepared at a 1:500 ratio of dye in BSA for 12 h at 4℃. Epithelial tumor tissue‐specific surface staining was performed with Alexa Fluor 594‐tagged variants of anti‐epithelial cell adhesion molecule (EpCAM, CD326; Biolegend) using a 1:300 dilution of dye in BSA and incubation for 2–3 days. Image acquisition was performed using confocal microscopy (Nikon Ti‐2) to produce slice and z‐stackable images of tumor spheroid and vasculature. ImageJ (http://fiji.sc.), an open‐access software, was used to examine the confocal images.

### 2.10 Statistical analyses

The data are presented as the mean ± SD. All statistical analyses were carried out using GraphPad Prism (GraphPad software). A *p* value <0.05 was considered statistically significant. All statistical tests were two‐sided.

## 3 RESULTS

### 3.1 Classification of the GC cell lines

**FIGURE 1:**
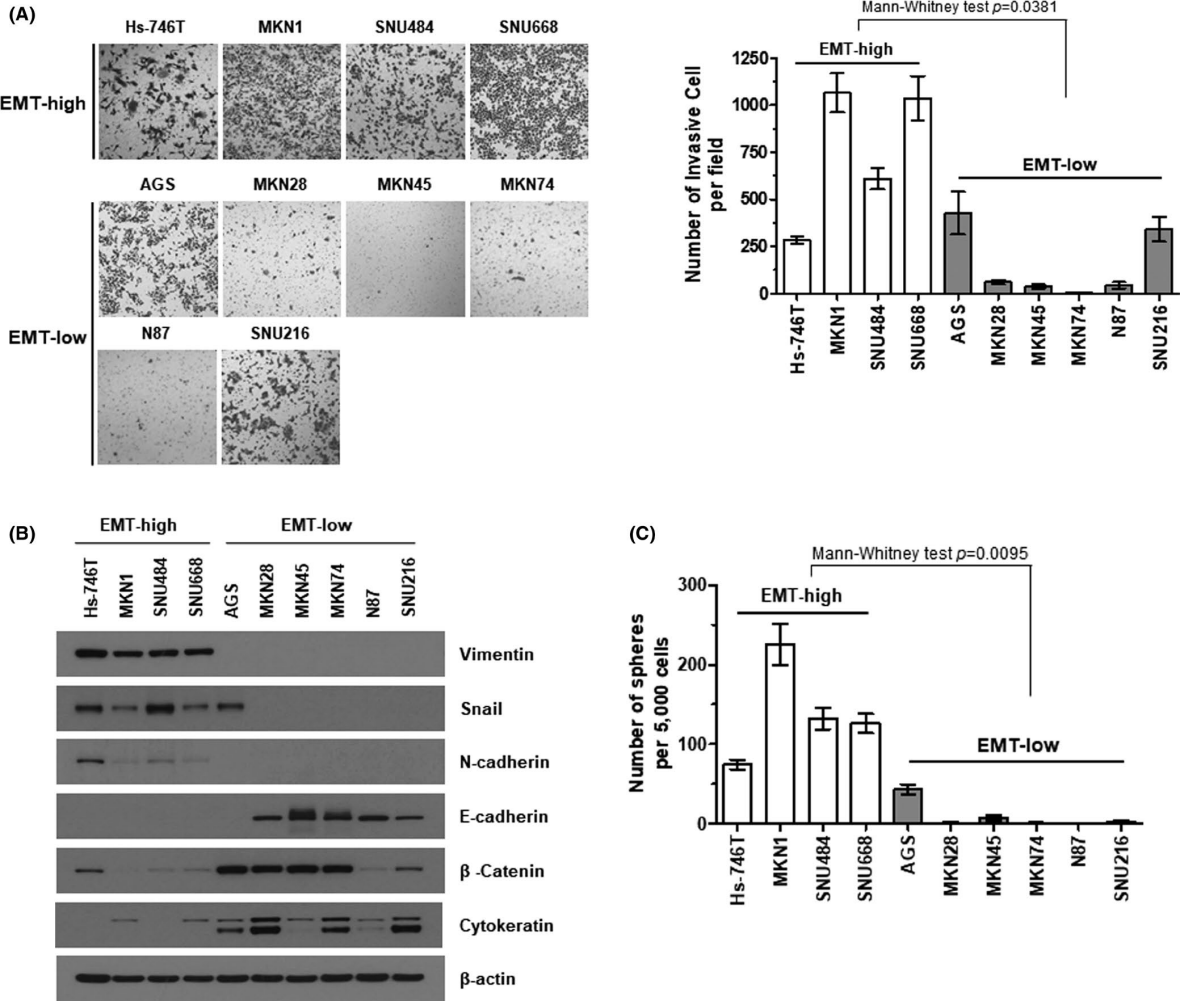
Classification of the GC cell lines. (A) Matrigel invasion assay. Representative bright‐field microscopic images of the cells that passed through Matrigel‐coated Transwell membranes are shown (*left*). The ability of each cell line to pass through Transwell membranes inserts was quantified (*right*). Statistical differences between the EMT‐high and EMT‐low groups were determined using Mann–Whitney test. (B) Confirmation of the EMT protein markers in EMT‐high and EMT‐low cells. Steady‐state expression of individual proteins was assessed by western blotting of the whole cell lysates from the indicated EMT‐high or EMT‐low GC cell lines. Beta‐actin was used as a loading control. (C) Tumor spheroid‐forming capabilities of gastric cancer cell lines. A spheroid‐forming colony was defined as a non‐adherent colony of cells derived from a single stem‐like cancer cell that was greater than 50 μm in diameter, and quantified. Statistical differences were determined using Mann–Whitney test

Prior to identifying antitumoral effect of drugs targeting diffuse‐type GC, we classified GC cell lines, in order to select cell lines showing high EMTness. This, in turn, could represent characteristics of the diffuse‐type GC. Out of 10 cancer cell lines tested, 4 cell lines (Hs‐746T, MKN1, SNU484, and SNU668) tended to migrate across the porous membranes more easily than did other 6 cell lines (AGS, MKN28, MKN45, MKN74, N87, and SNU216) in an invasion assay which evaluates passage of GC cells using a Matrigel Transwell system (Figure 1A). We classified Hs‐746T, MKN1, SNU484, and SNU668 as an EMT‐high group and the others as an EMT‐low. It is well known that, as the EMT progresses, expression of epithelial marker proteins including E‐cadherin and β‐catenin is reduced, and epithelial cells increase the expression of mesenchymal markers such as N‐cadherin and vimentin, which results in invasive phenotypes.^18^ Each subtype of the cells we assessed generally followed this same pattern of marker expression (Figure 1B). In addition, when grown in ultra‐low attachment plates that enable cells to form three dimensional spheres, the EMT‐high cells produced a larger number of tumor spheroids than did the EMT‐low cells (Figure 1C), demonstrating that cells classified into the EMT‐high had stem cell‐like features that are thought to contribute to tumor progression and metastasis.^19^ The line characterization in our study was in accordance with previous results.^20^

### 3.2 Pharmacological inhibitors regulating transforming growth factor β (TGF‐β) receptor kinase and an anti‐VEGF receptor 2 antibody, ramucirumab, alleviate invasiveness of the EMT‐high GC cell lines

**FIGURE 2:**
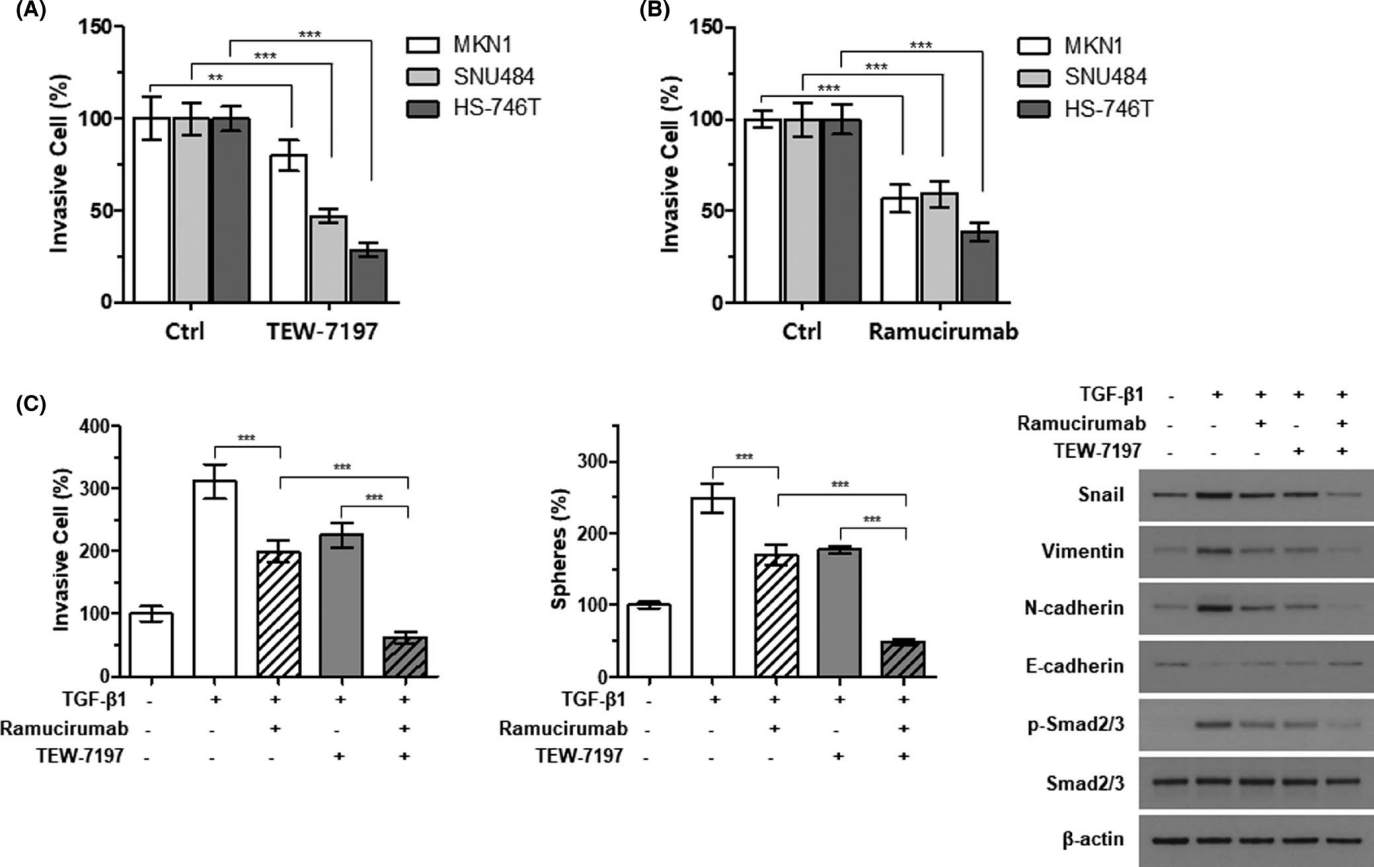
Synergistic effect of TEW‐7197 and ramucirumab on the EMT‐high GC cell lines. The effects of TGF‐β receptor inhibitor, TEW‐7197 (A) and anti‐VEGF receptor 2 antibody, ramucirumab (B) on invasiveness of the EMT‐high GC cell lines. The invasive capabilities of each cell line were estimated with a Matrigel Transwell assay after 48 h treatment with 3 μM TEW‐7179 (A) or 3 μM ramucirumab (B). Equal numbers of the indicated cells were loaded onto the membrane insert, and the number of cells passing through the pores was quantified. Statistical differences between control (Ctrl) and treated were determined using two‐way ANOVA with Bonferroni‐adjusted post hoc tests for multiple comparisons. (C) SNU484 cells were incubated with 5 ng/ml of TGF‐β in the absence or presence of 2 μM ramucirumab and/or 1 μM TEW‐7179 for 48 h. The number of cells passing through the membrane inserts was determined with a Matrigel Transwell assay (*left*), and a spheroid‐forming colony was quantified as described before (*middle*). The expression of EMT markers was detected by immunoblotting (*right*). Beta‐actin was used as a loading control. Statistical differences were determined using one‐way ANOVA with Bonferroni‐adjusted post hoc tests for multiple comparisons. **p < 0.01, ***p < 0.001

Given that the EMT process shares many aspects with signaling pathways involved in stem cell regulation,^21^ we sought a drug candidate that could regulate the EMT phenotype of cancer cells from the following known stem cell signaling inhibitors such as TGF‐β receptor kinase inhibitors (TEW‐7197 and LY3200882). For TGF‐β receptor kinase inhibitors, we pre‐treated the cells lines with TGF‐β ligands to directly activate TGF‐β signaling and minimize potential secondary effects and confirmed activation of TGF‐β signaling with phosphorylated Smad2/3 levels. Then we investigated whether the invasiveness of the EMT‐high cells was affected by the inhibitors. TEW‐7197 and LY3200882 both considerably decreased the invasiveness of EMT‐high GC cell lines (Figure 2A and Supplementary Figure S1). In addition, ramucirumab significantly reduced invasiveness of the EMT‐high GC cell lines (Figure 2B). While the effect of VEGF or VEGFR on angiogenesis is well‐known, only a few studies demonstrated its influence on proliferation, migration, or invasion in cancer cells.^22^, ^23^, ^24^ Considering a pleiotropic role of TGF‐β signaling on tumorigenesis, such as cancer cell proliferation, metastasis, chemoresistance, even on angiogenesis^25^, ^26^, ^27^ and based on a fact that VEGF induces the EMT and cancer stemness through cross talk with TGF‐β,^28^, ^29^ we further investigated synergism between TGF‐β receptor inhibitor, TEW‐7197, and ramucirumab. In the invasion assay with one of the EMT‐high cell lines, SNU484, incubating the cells with TEW‐7197 plus ramucirumab had a greater influence on invasion and spheroid‐forming capabilities of the cells than did either inhibitor alone (*left* and *middle* columns of Figure 2C, respectively). Another EMT‐high cell line, MKN1, reproduced similar results when incubated with TEW‐7197 and ramucirumab together (Supplementary Figure S2). Western blot assays demonstrated the additive effects of two inhibitors on EMT status (*right* column of Figure 2C).

### 3.3 GC patient‐derived cells (PDCs) cultured from malignant ascites of diffuse‐type GC are sensitive to ramucirumab plus TGF‐β receptor kinase inhibitor

**FIGURE 3:**
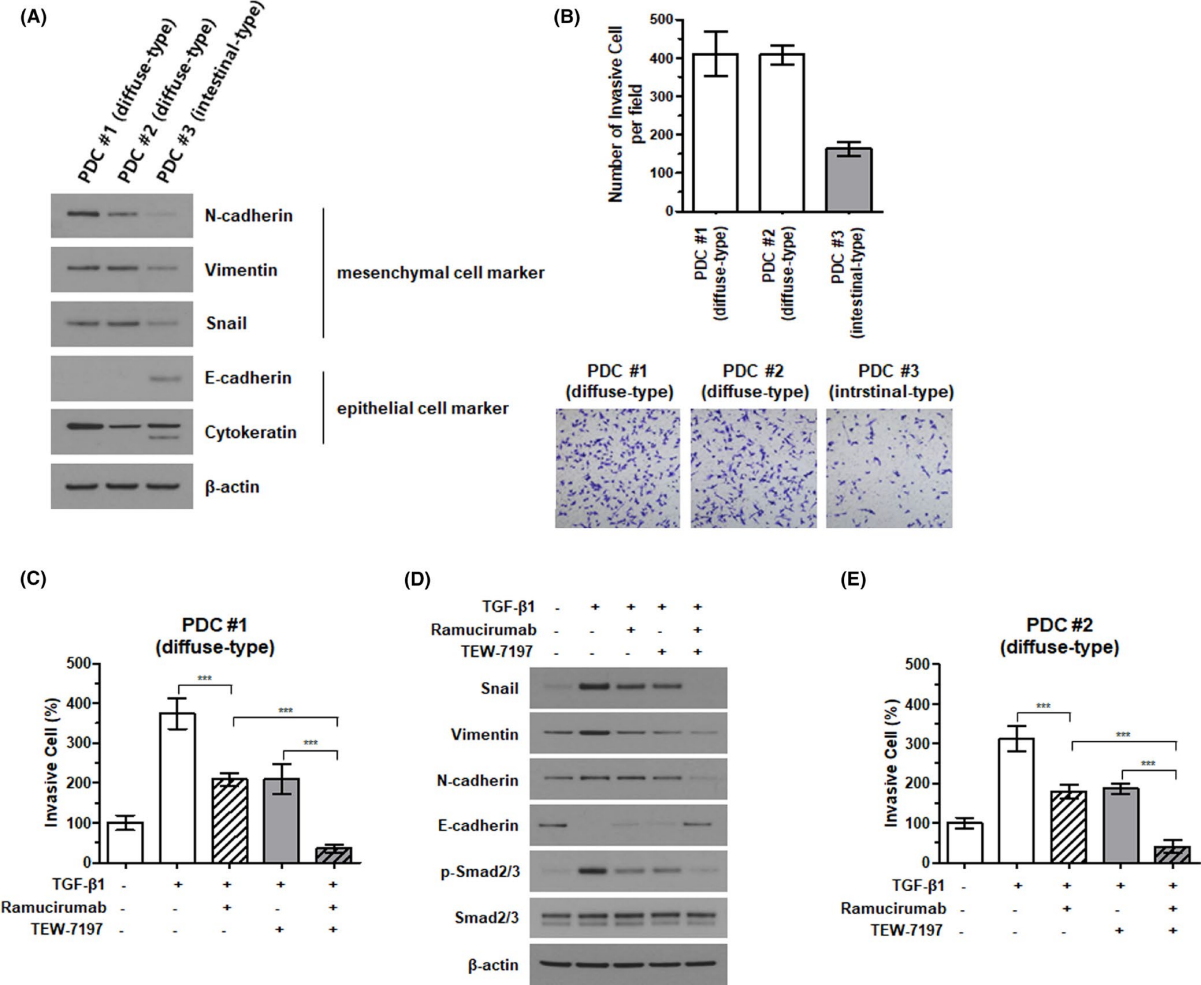
Synergistic effects of TEW‐7197 and ramucirumab on gastric cancer patient‐derived cells (PDCs). Classification of EMT characteristics in gastric cancer patient‐derived cells (PDCs). PDCs from three gastric cancer patients were used to assess the expression of mesenchymal or epithelial cell protein markers (A) and the invasiveness (B). Each PDC was treated with 10 ng/ml of TGF‐β in the absence or presence of 2 μM ramucirumab and/or 1 μM TEW‐7179 for 48 h, and the invasion assay was conducted (C and E). Statistical differences were determined using one‐way ANOVA with Bonferroni‐adjusted post hoc tests for multiple comparisons. ***p < 0.001. Western blotting of the diffuse‐type PDC #1 was performed to evaluate the expression of EMT marker proteins (D)

Next, we investigated EMTness of GC PCDs. PDC #1 was cultured from malignant cells isolated from a 37‐year‐old female GC patient (EBV negative, HER2 negative, mismatch repair proficient, and signet ring cell carcinoma). PDC #2 was cultured from malignant cells isolated from a 55‐year‐old female GC patient (EBV negative, HER2 negative, mismatch repair proficient, and signet ring cell carcinoma). PDC #3 was cultured from malignant cells isolated from a 42‐year‐old male GC patient (EBV negative, HER2 negative, mismatch repair proficient, and tubular adenocarcinoma/intestinal type). Even though the expression patterns of epithelial or mesenchymal protein markers in PDCs were not as apparent as those of the GC cell lines (Figure 1B and Figure 3A), of three PDCs, two that once were isolated from the diffuse‐type GC displayed greater expression of mesenchymal markers, such as N‐cadherin, vimentin, and SNAIL. The diffuse‐type PDCs (PDC #1 and PDC #2) had greater penetration capability through the Transwell inserts (Figure 3B) and tended to form a larger number of tumor spheroids than the intestinal‐type PDC (PDC #3) (Supplementary Figure S3). Incubating the PDCs with TEW‐7197 or ramucirumab alone had only marginal effects on invasiveness and spheroid formation of the cells. However, co‐incubation with the two agents efficiently reduced the number of cells reaching the lower chambers of the Transwell plates and tumor spheroid formation (Figure 3C, 3E and Supplementary Figure S4, respectively). Western blot analyses confirmed that ramucirumab together with TEW‐7197 reduced the expression of mesenchymal markers while increasing the expression of epithelial markers (Figure 3D).

### 3.4 The addition of TGF‐β receptor kinase inhibitor to ramucirumab blocks blood vessel formation in a PDC model

**FIGURE 4:**
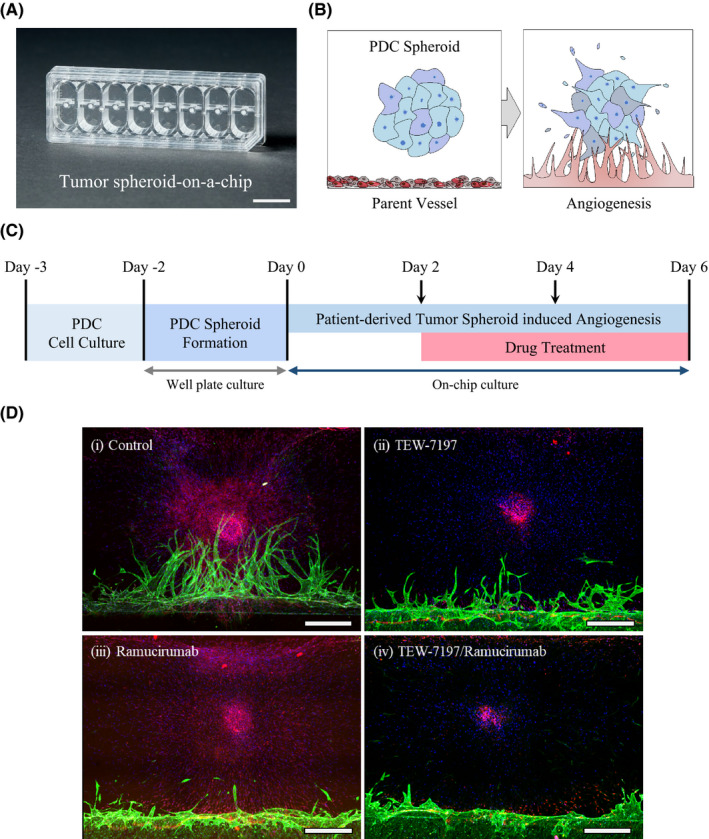
Validation of drug efficacy using tumor spheroid‐induced 3D angiogenesis assay derived from diffuse‐type PDC representing high EMTness. (A) The photograph of the standardized tumor spheroid‐on‐a‐chip capable of mimicking tumor microenvironment. Scale bar: 1 mm. (B) Schematic of 3D angiogenesis induced by a patient‐derived tumor spheroid placed within an ECM. (C) Timeline for PDC spheroid‐induced angiogenesis and drug treatment. (D) Confocal 3D projection images showing diffuse‐type PDC #1 spheroid‐induced angiogenesis. Various types of drugs were treated on the chip. (i) Control, (ii) TEW‐7197, (iii) ramucirumab, and (iv) TEW‐7197 with ramucirumab. Green: Lectin (Alexa Fluor 488), Red: EpCAM (Alexa Fluor 594), and Blue: DAPI. Scale bar: 500 μm

We utilized a tumor spheroid‐on‐a‐chip capable of 3D blood vessel formation to evaluate the effect of co‐administration of ramucirumab and TEW‐7197. Previously, Ko et al.^17^ developed the tumor spheroid‐on‐a‐chip, an in vitro 3D cell culture chip that can recapitulate the tumor microenvironment including tumor and blood vessels (Figure 4A). This chip provides 3D image data of the morphological features of tumors and blood vessels that can explicate drug response effects visually.

We conducted PDC spheroid‐induced 3D angiogenesis experiments using diffuse‐type PDC #1. Before on‐chip culture, the PDC spheroid was formed for 2 days in U‐shaped 96‐well plates. This spheroid was patterned three‐dimensionally on the chip's cell culture channel with fibrin hydrogel and fibroblast. And then, endothelial cells were placed on the primed hydrogel interface to form an endothelialized layer. This layer acts as a parent vessel, inducing vasculogenesis toward the tumor spheroid (Figure 4B). After loading the tumor cells, angiogenesis was established 6 days later. During the process, we contributed the difference in the media level to generate flow conditions. This condition allowed the various growth factors secreted from the PDC spheroid to initiate the parent vessel sprouting. Using this model, we evaluated the effect of drug treatment on angiogenesis induced by PDC spheroid. Notably, the blood vessel formation with diffuse‐type PDC‐EMT #1 was inhibited by ramucirumab to a great extent, as well as by TEW‐7197 (Figure 4D). Furthermore, aberrant morphological characteristics were exhibited from the tip cell, and the tip cell looked lumpy. This antiangiogenic effect can also be a strategy for treating diffuse‐type GC patients with poor prognosis as a synergistic effect by lowering resistance, and at the same time, by interfering nutrient and oxygen supply through combination therapy.

## 4 DISCUSSION

In this study, we identified that inhibition of VEGF receptor or TGF‐β receptor signaling pathway with ramucirumab or TEW‐7197, respectively, alleviated EMTness of diffuse‐type GC cells. TGF‐β pathway, as one of the well‐known EMT inducers, activates the expression of mesenchymal genes such as *SNAIL* and *ZEB1* through a SMAD family dependent cascade and SMAD‐independent MAPK/PI3K/AKT signaling pathways.^30^, ^31^ It has been reported that high levels of TGF‐β in tumor microenvironment and plasma were correlated with cancer progression, metastasis, and poor prognosis^32^, ^33^, ^34^ which is rather attributed from TGF‐β‐mediated chemoresistance.^35^, ^36^, ^37^

Despite most of the researches with ramucirumab focused on its ability to regulate endothelial cell signaling, we demonstrated that ramucirumab reduced the invasive capability of EMT‐high GC cell lines (Figure 2B), suggesting VEGF receptor as a candidate for EMT‐targeted therapy. Combining ramucirumab with TGF‐β receptor kinase inhibitor, TEW‐7197, produced additive effects, significantly reducing the invasion and spheroid‐forming capabilities of EMT‐high cell lines (SNU484 and MKN1) compared with either the inhibitor or ramucirumab alone. In fact, some reports pointed out that antiangiogenic agents could contribute to anti‐tumorigenesis by directly targeting the stemness of tumor cells, not just by controlling vascular structure.^38^ In addition, Von Baumgarten et al. showed dose‐dependent effects of another antiangiogenic monoclonal antibody, bevacizumab, on neoplastic cell viability and induction of cytotoxicity in vivo.^39^ We confirmed the additive effects of TEW‐7197 to ramucirumab in PDC models. Incubation with the two agents reduced invasiveness and spheroid formation of the PDCs to a greater degree than either inhibitor alone. Western blot analyses revealed that combining the two agents also reduced mesenchymal (SNAIL, vimentin, and N‐cadherin) and induced epithelial (E‐cadherin) marker expression, suggesting reversal of the EMT in treated cell lines. These findings were consistent with those of a previous report evaluating the effects of a combination of ramucirumab and paclitaxel in GC cell lines, in which E‐cadherin was upregulated and N‐cadherin was downregulated.^40^ Hence ramucirumab and TGF‐β receptor kinase inhibitor may profoundly reduce invasiveness of GC cells, especially those with high EMT features, such as malignant cells from diffuse‐type GC ascites.

We also validated a direct action of ramucirumab on endothelial cells with the tumor spheroid‐on‐a‐chip which enabled us to captivate blood vessel formation induced by cancer cell spheroid (Figure 4). Treatment with ramucirumab at the initial point of angiogenesis revealed a noticeable decline in the vessel sprouting. Ramucirumab, which binds to VEGFR2, is one of the most effective treatments for inhibiting blood vessel formation in GC patients. During combination therapy using antiangiogenic agents with chemotherapeutics, the tumor vasculature would change depending on pro‐ and antiangiogenic signals. This could lead to increased blood perfusion and chemotherapy delivery or to vessel collapse and consequent limited nutrients and oxygen supply.^41^, ^42^ Thus, it is important to precisely explore therapy influence on vascular structure, drug delivery, and growth/regression of tumor cells. In respect of this, the 3D cell culture platform we used would provide us an integrated view to apprehend tumor microenvironment by examining interaction between cancer cells and surrounding blood vessels.

Taken together, our study confirmed that combination therapy with simultaneous use of TEW‐7197 and ramucirumab exerted additive effects on reversing the EMT, in addition to interfering the tumor‐induced angiogenesis, of diffuse‐type GC in an in vitro model. Moreover, we demonstrated for the first time that a 3D tumor spheroid‐on‐a‐chip can be efficiently used to assess angiogenesis with patient‐derived cell lines. We plan to apply this 3D tumor spheroid‐on‐a‐chip in various applications in a more high‐throughput drug screening system.

## CONFLICTS OF INTEREST

The authors declare no competing interests.

## AUTHOR CONTRIBUTIONS

Conception and design: Jeeyun Lee, Song‐Yi Lee, Seonggyu Byeon, and Jung Yong Hong; Development of methodology: Jihoon Ko, Sujin Hyung, Noo Li Jeon, and Jeeyun Lee; Acquisition of data: Jihoon Ko, Sujin Hyung, In‐Kyoung Lee, Se Hoon Park, Jung Yong Hong, and Jeeyun Lee; Analysis and interpretation of data: Jeeyun Lee, Song‐Yi Lee, Seonggyu Byeon, Jihoon Ko, Sujin Hyung, and Jung Yong Hong; Writing, reviewing, and/or revising the manuscript: Song‐Yi Lee, Seonggyu Byeon, Jihoon Ko, and Jeeyun Lee; Study supervision: Jeeyun Lee.

## ETHICAL APPROVAL

The study was approved by the committee of Samsung Medical Center (SMC), Seoul, Republic of Korea, on the use of human samples for experimental studies. Written informed consent was obtained from all study participants prior to enrollment. The research conformed to the principles of the Helsinki Declaration.

## CONSENT FOR PUBLICATION

Written informed consent was obtained from all study participants prior to enrollment and they also provided consent for publication.

## Supporting information

Fig S1

Fig S2

Fig S3

Fig S4

## Data Availability

The data that support the findings of this study are available from the corresponding author upon reasonable request.

## References

[1] Fitzmaurice C , Akinyemiju TF , Al Lami FH , et al. Global, regional, and national cancer incidence, mortality, years of life lost, years lived with disability, and disability‐adjusted life‐years for 29 cancer groups, 1990 to 2016. JAMA Oncology. 2018;4:1553. 29860482 10.1001/jamaoncol.2018.2706 PMC6248091

[2] de Vries AC , van Grieken NCT , Looman CWN , et al. Gastric cancer risk in patients with premalignant gastric lesions: a nationwide cohort study in the Netherlands. Gastroenterology. 2008;134:945‐952. 18395075 10.1053/j.gastro.2008.01.071

[3] Graham DY . *Helicobacter pylori* update: gastric cancer, reliable therapy, and possible benefits. Gastroenterology. 2015;148:719‐731.e3. 25655557 10.1053/j.gastro.2015.01.040 PMC4375058

[4] Zhang X , Li M , Chen S , et al. Endoscopic screening in Asian countries is associated with reduced gastric cancer mortality: a meta‐analysis and systematic review. Gastroenterology. 2018;155:347‐354.e9. 29723507 10.1053/j.gastro.2018.04.026

[5] Marqués‐Lespier JM , González‐Pons M , Cruz‐Correa M . Current perspectives on gastric cancer. Gastroenterol Clin North Am. 2016;45:413‐428. 27546840 10.1016/j.gtc.2016.04.002 PMC4993977

[6] Genome Atlas Research Network . Comprehensive molecular characterization of gastric adenocarcinoma. Nature. 2014;513:202‐209. 25079317 10.1038/nature13480 PMC4170219

[7] Cristescu R , Lee J , Nebozhyn M , et al. Molecular analysis of gastric cancer identifies subtypes associated with distinct clinical outcomes. Nat Med. 2015;21:449‐456. 25894828 10.1038/nm.3850

[8] Tsai JH , Yang J . Epithelial‐mesenchymal plasticity in carcinoma metastasis. Genes Dev. 2013;27:2192‐2206. 24142872 10.1101/gad.225334.113 PMC3814640

[9] Peng Z . Role of epithelial‐mesenchymal transition in gastric cancer initiation and progression. World J Gastroenterol. 2014;20:5403. 24833870 10.3748/wjg.v20.i18.5403 PMC4017055

[10] Izumiya M , Kabashima A , Higuchi H , et al. Chemoresistance is associated with cancer stem cell‐like properties and epithelial‐to‐mesenchymal transition in pancreatic cancer cells. Anticancer Res. 2012;32:3847‐3853. 22993328

[11] Grygielewicz P , Dymek B , Bujak A , et al. Epithelial–mesenchymal transition confers resistance to selective FGFR inhibitors in SNU‐16 gastric cancer cells. Gastric Cancer. 2016;19:53‐62. 25407459 10.1007/s10120-014-0444-1 PMC4688307

[12] Selim JH , Shaheen S , Sheu W‐C , et al. Targeted and novel therapy in advanced gastric cancer. Experimental Hematology & Oncology. 2019;8. 10.1186/s40164-019-0149-6 PMC6788003 31632839

[13] Wilke H , Muro K , Van Cutsem E , et al. Ramucirumab plus paclitaxel versus placebo plus paclitaxel in patients with previously treated advanced gastric or gastro‐oesophageal junction adenocarcinoma (RAINBOW): a double‐blind, randomised phase 3 trial. Lancet Oncol. 2014;15:1224‐1235. 25240821 10.1016/S1470-2045(14)70420-6

[14] Hwang J‐E , Lee J‐H , Park M‐R , et al. Blockade of VEGFR‐1 and VEGFR‐2 enhances paclitaxel sensitivity in gastric cancer cells. Yonsei Med J. 2013;54:374. 23364970 10.3349/ymj.2013.54.2.374 PMC3575962

[15] Lee J‐K , Liu Z , Sa JK , et al. Pharmacogenomic landscape of patient‐derived tumor cells informs precision oncology therapy. Nat Genet. 2018;50:1399‐1411. 30262818 10.1038/s41588-018-0209-6 PMC8514738

[16] Cho JH , Kim J‐S , Kim ST , et al. Selective colony area method for heterogeneous patient‐derived tumor cell lines in anti‐cancer drug screening system. PLoS One. 2019;14:e0215080. 30995234 10.1371/journal.pone.0215080 PMC6469764

[17] Ko J , Ahn J , Kim S , et al. Tumor spheroid‐on‐a‐chip: a standardized microfluidic culture platform for investigating tumor angiogenesis. Lab Chip. 2019;19:2822‐2833. 31360969 10.1039/c9lc00140a

[18] Cho ES , Kang HE , Kim NH , et al. Therapeutic implications of cancer epithelial‐mesenchymal transition (EMT). Arch Pharm Res. 2019;42:14‐24. 30649699 10.1007/s12272-018-01108-7

[19] Sampieri K , Fodde R . Cancer stem cells and metastasis. Semin Cancer Biol. 2012;22:187‐193. 22774232 10.1016/j.semcancer.2012.03.002

[20] Lee J , Kim H , Lee JE , et al. Selective cytotoxicity of the NAMPT inhibitor FK866 toward gastric cancer cells with markers of the epithelial‐mesenchymal transition, due to loss of NAPRT. Gastroenterology. 2018;155:799‐814.e13. 29775598 10.1053/j.gastro.2018.05.024

[21] Abell AN , Johnson GL . Implications of mesenchymal cells in cancer stem cell populations: relevance to EMT. Curr Pathobiol Rep. 2014;2:21‐26. 25530923 10.1007/s40139-013-0034-7 PMC4266994

[22] Lian L , Li X‐L , Xu M‐D , et al. VEGFR2 promotes tumorigenesis and metastasis in a pro‐angiogenic‐independent way in gastric cancer. BMC Cancer. 2019;19. 10.1186/s12885-019-5322-0 PMC6393973 30819137

[23] Lin J‐Z , Wang Z‐J , De W , et al. Targeting AXL overcomes resistance to docetaxel therapy in advanced prostate cancer. Oncotarget. 2017;8:41064‐41077. 28455956 10.18632/oncotarget.17026 PMC5522277

[24] Morelli MP , Brown AM , Pitts TM , et al. Targeting vascular endothelial growth factor receptor‐1 and ‐3 with cediranib (AZD2171): effects on migration and invasion of gastrointestinal cancer cell lines. Mol Cancer Ther. 2009;8:2546‐2558. 19755510 10.1158/1535-7163.MCT-09-0380 PMC2819052

[25] Ikushima H , Miyazono K . TGFβ signalling: a complex web in cancer progression. Nat Rev Cancer. 2010;10:415‐424. 20495575 10.1038/nrc2853

[26] Massagué J . TGFβ signalling in context. Nat Rev Mol Cell Biol. 2012;13:616‐630. 22992590 10.1038/nrm3434 PMC4027049

[27] Goumans M‐J , Liu Z , Ten Dijke P . TGF‐β signaling in vascular biology and dysfunction. Cell Res. 2009;19:116‐127. 19114994 10.1038/cr.2008.326

[28] Gonzalez‐Moreno O , Lecanda J , Green JE , et al. VEGF elicits epithelial‐mesenchymal transition (EMT) in prostate intraepithelial neoplasia (PIN)‐like cells via an autocrine loop. Exp Cell Res. 2010;316:554‐567. 20006606 10.1016/j.yexcr.2009.11.020

[29] Fantozzi A , Gruber DC , Pisarsky L , et al. VEGF‐mediated angiogenesis links EMT‐induced cancer stemness to tumor initiation. Can Res. 2014;74:1566. 10.1158/0008-5472.CAN-13-1641 24413534

[30] Derynck R , Muthusamy BP , Saeteurn KY . Signaling pathway cooperation in TGF‐β‐induced epithelial‐mesenchymal transition. Curr Opin Cell Biol. 2014;31:56‐66. 25240174 10.1016/j.ceb.2014.09.001 PMC4657734

[31] Zhang J , Tian XJ , Xing J . Signal transduction pathways of EMT induced by TGF‐β, SHH, and WNT and their crosstalks. J Clin Med 2016;5(4):41. 10.3390/jcm5040041 PMC4850464 27043642

[32] Hasegawa Y , Takanashi S , Kanehira Y , et al. Transforming growth factor‐beta1 level correlates with angiogenesis, tumor progression, and prognosis in patients with nonsmall cell lung carcinoma. Cancer. 2001;91:964‐971. 11251948

[33] Desruisseau S , Palmari J , Giusti C , et al. Determination of TGFβ1 protein level in human primary breast cancers and its relationship with survival. Br J Cancer. 2006;94:239‐246. 16404434 10.1038/sj.bjc.6602920 PMC2361106

[34] Li J , Shen C , Wang X , et al. Prognostic value of TGF‐β in lung cancer: systematic review and meta‐analysis. BMC Cancer. 2019;19. 10.1186/s12885-019-5917-5 PMC6631541 31307405

[35] Mao L , Li Y , Zhao J , et al. Transforming growth factor‐β1 contributes to oxaliplatin resistance in colorectal cancer via epithelial to mesenchymal transition. Oncology Letters. 2017;14:647‐654. 28693217 10.3892/ol.2017.6209 PMC5494727

[36] Katsuno Y , Meyer DS , Zhang Z , et al. Chronic TGF‐β exposure drives stabilized EMT, tumor stemness, and cancer drug resistance with vulnerability to bitopic mTOR inhibition. Science Signaling 2019;12:eaau8544. 30808819 10.1126/scisignal.aau8544 PMC6746178

[37] Bhagyaraj E , Ahuja N , Kumar S , et al. TGF‐β induced chemoresistance in liver cancer is modulated by xenobiotic nuclear receptor PXR. Cell Cycle. 2019;18:3589‐3602. 31739702 10.1080/15384101.2019.1693120 PMC6927732

[38] Beck B , Driessens G , Goossens S , et al. A vascular niche and a VEGF–Nrp1 loop regulate the initiation and stemness of skin tumours. Nature. 2011;478:399‐403. 22012397 10.1038/nature10525

[39] von Baumgarten L , Brucker D , Tirniceru A , et al. Bevacizumab has differential and dose‐dependent effects on glioma blood vessels and tumor cells. Clin Cancer Res. 2011;17:6192‐6205. 21788357 10.1158/1078-0432.CCR-10-1868

[40] Refolo MG , Lotesoriere C , Lolli IR , et al. Molecular mechanisms of synergistic action of ramucirumab and paclitaxel in gastric cancers cell lines. Sci Rep. 2020;10:7162. 32346056 10.1038/s41598-020-64195-x PMC7188894

[41] Jain RK . Antiangiogenesis strategies revisited: from starving tumors to alleviating hypoxia. Cancer Cell. 2014;26:605‐622. 25517747 10.1016/j.ccell.2014.10.006 PMC4269830

[42] Semenza GL . Oxygen sensing, hypoxia‐inducible factors, and disease pathophysiology. Annu Rev Pathol. 2014;9:47‐71. 23937437 10.1146/annurev-pathol-012513-104720

