# Supplementary figures and images for: Reducing tumor invasiveness by ramucirumab and TGF‐β receptor kinase inhibitor in a diffuse‐type gastric cancer patient‐derived cell model
Source: Cancer Med. 2021 Sep 20;10(20):7253–62. doi: 10.1002/cam4.4259 (PMC8525100; doi:10.1002/cam4.4259)

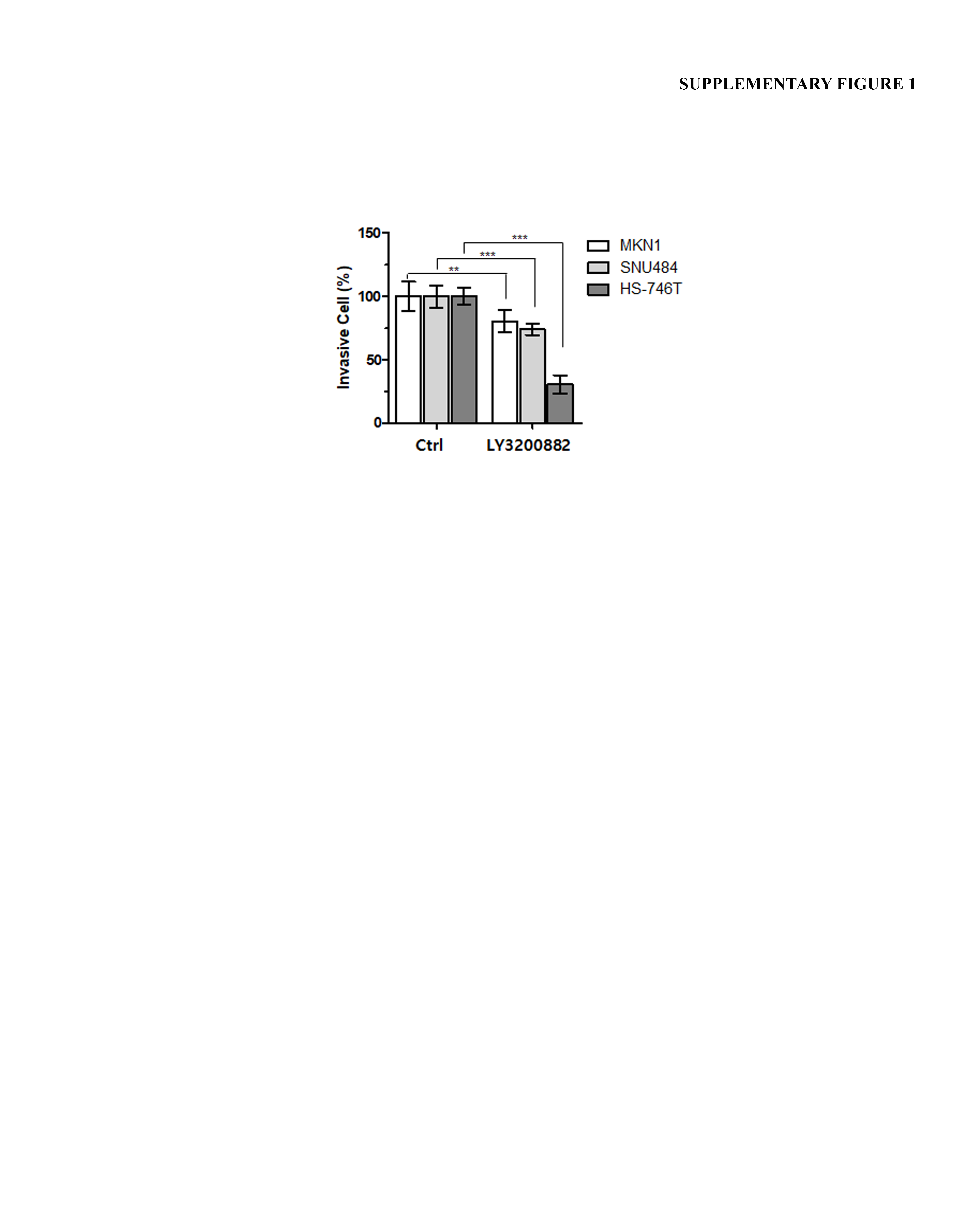

Supplement: Supplementary file 1 — Fig S1 [file CAM4-10-7253-s004.tif]

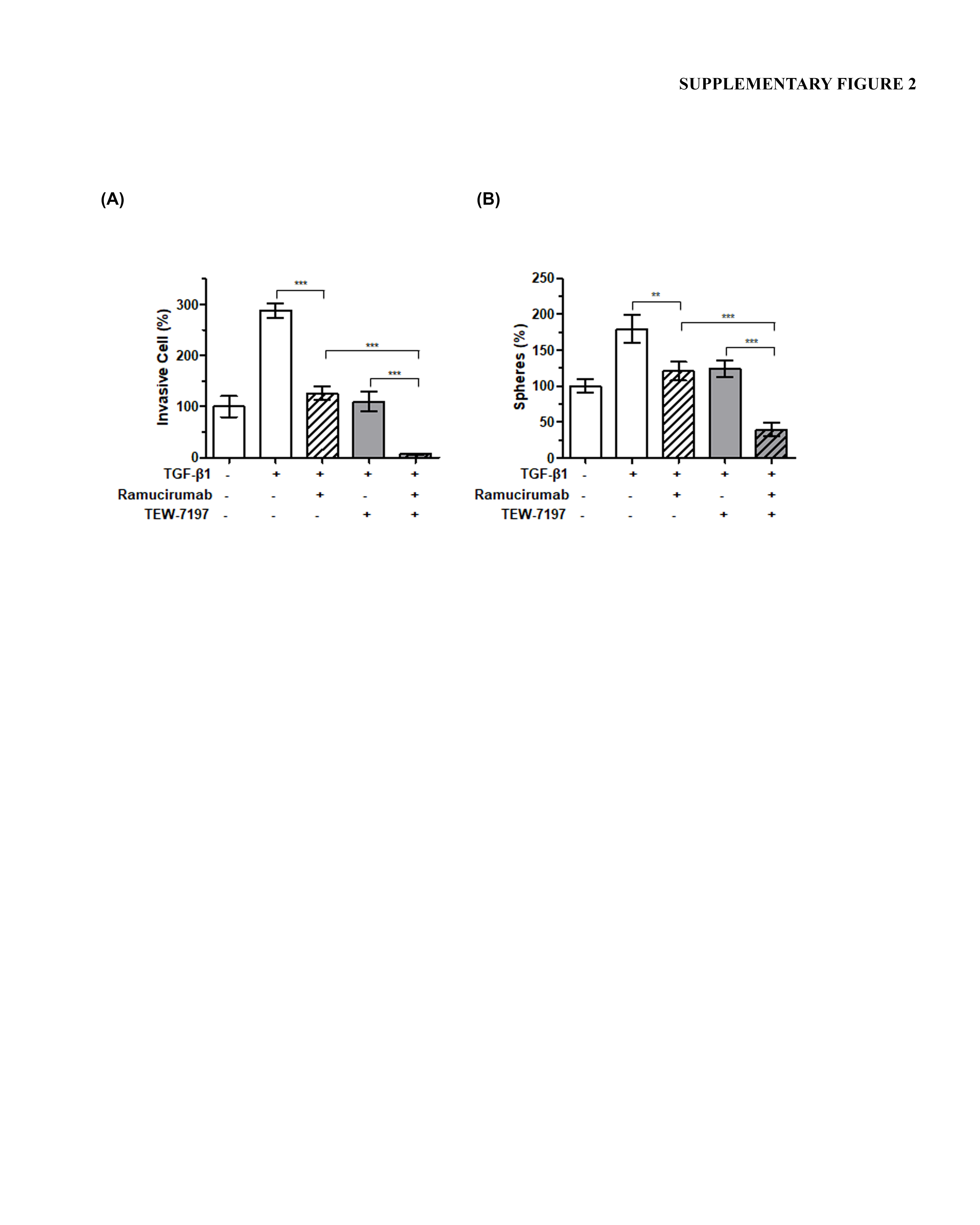

Supplement: Supplementary file 2 — Fig S2 [file CAM4-10-7253-s001.tif]

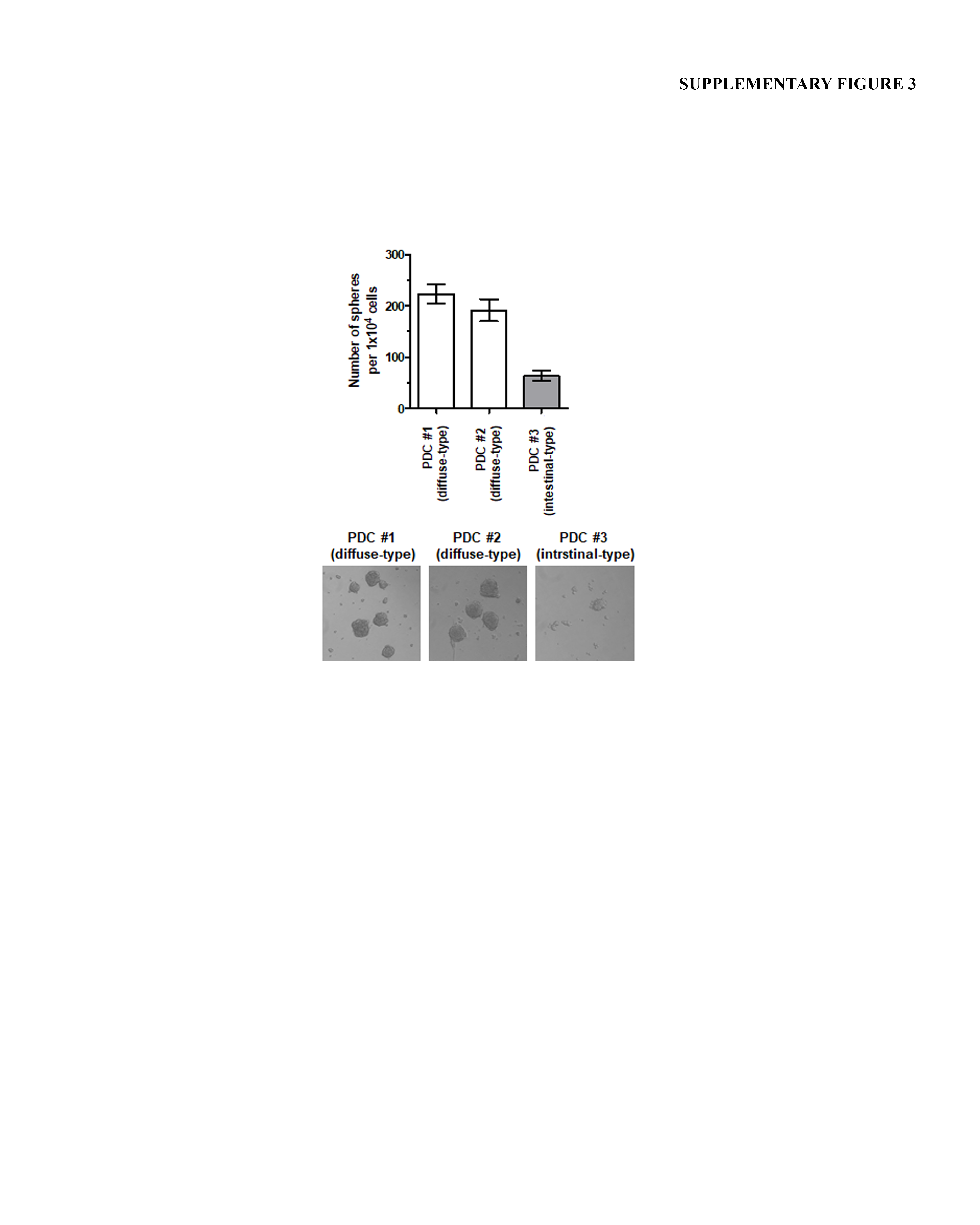

Supplement: Supplementary file 3 — Fig S3 [file CAM4-10-7253-s002.tif]

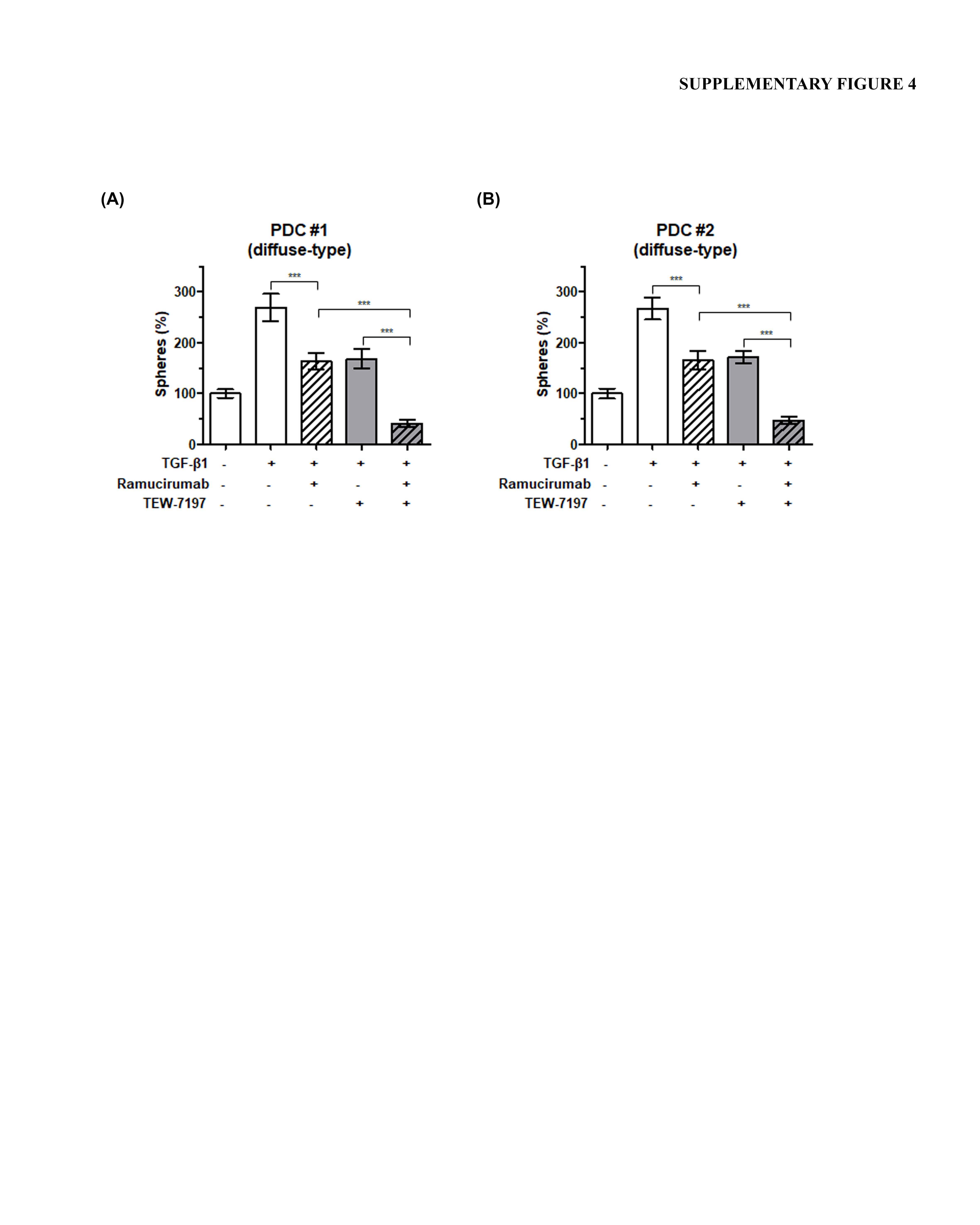

Supplement: Supplementary file 4 — Fig S4 [file CAM4-10-7253-s003.tif]
